# Propeptide genesis by Kex2-dependent cleavage of yeast wall protein 1 (Ywp1) of *Candida albicans*

**DOI:** 10.1371/journal.pone.0207955

**Published:** 2018-11-26

**Authors:** Bruce L. Granger

**Affiliations:** Department of Microbiology and Immunology, Montana State University, Bozeman, Montana, United States of America; Louisiana State University, UNITED STATES

## Abstract

*Candida albicans* is a prevalent fungal resident and opportunistic pathogen of humans, exhibiting a variety of ovoid and filamentous morphologies. Anchored within the cell wall of the ovoid yeast form of *C*. *albicans* is an abundant glycoprotein termed yeast wall protein 1 (Ywp1). Ywp1 has an antiadhesive effect that may facilitate yeast cell dispersal; it also contributes to the masking of the glucan matrix of the yeast cell wall, potentially providing shielding from recognition by the human immune system. Mature Ywp1 consists of an O-glycosylated core of 378 amino acids associated with an N-glycosylated propeptide that originates from an N-terminal segment of Ywp1. A tribasic (-RRR-) sequence in the immature Ywp1 polypeptide is separated by 8 amino acids from a dibasic (-KR-) sequence that is a canonical site for cleavage by the intracellular endopeptidase Kex2, and cleavage occurs at both of these sites to generate an 11 kilodalton (kDa) propeptide that remains strongly associated with the mature core of Ywp1. Previous studies demonstrated an absence of the 11 kDa propeptide in strains lacking Kex2, but the presence of lesser amounts of a 12 kDa propeptide ostensibly (and paradoxically) arising from cleavage at the dibasic site. Subsequent studies of wild type strains, however, suggested that post-secretion cleavages were carried out *in vitro* by acid proteases in unbuffered cultures to generate the 12 kDa propeptide. Here, intact and Gfp-tagged Ywp1 are utilized to show that neither of the two multibasic sites is normally cleaved in the absence of Kex2, but that uncleaved Ywp1 is still N-glycosylated and subsequently anchored to the cell wall. This furthers our understanding of the multistep cleavage of this highly conserved sequence, as well as the possible contributions of the cleaved propeptide to the maturation and functioning of Ywp1.

## Introduction

The prevalent commensalism of *Candida albicans* contributes to its predominance as a fungal pathogen of humans, particularly when immune defenses are compromised [1, 2]. *C*. *albicans* cells are capable of growing at most anatomical sites, from epithelial and mucosal surfaces to deep tissues, and the cells can assume a variety of morphologies, from separate ovoid to linked filamentous [3]. Each cell is surrounded by a polysaccharide-rich envelope, termed the cell wall, that is dynamic, durable and protective [4]. The inner layer of the cell wall is rich in glucan chains, and the outer layer, facing the environment and directly interacting with host tissues, is rich in covalently anchored glycoproteins [5]. One of the most abundant glycoproteins of the ovoid, yeast morphology of *C*. *albicans* is Ywp1 (yeast wall protein 1). Its presence in the wall decreases cellular adhesivity, suggesting a role in yeast dispersal [6, 7]. In addition, it contributes to the masking of cell wall glucans [8], which are recognized as threatening by the human innate immune system [9, 10]. The molecular mechanisms of these phenotypic characteristics of Ywp1 remain to be elucidated, but are presumably a function of the amino acid sequence, which exhibits a high degree of conservation among *Candida* species [6].

Based on direct analyses of Ywp1 protein and predictions from its encoding gene sequence, the 533 amino acid Ywp1 polypeptide begins synthesis on cytosolic ribosomes, with the N-terminal 21 amino acid signal peptide directing translocation into the secretory pathway before being excised and presumably degraded. A single N-glycan is cotranslationally added to asparagine115; that glycan is subsequently modified, especially by addition of multiple mannose units, while shorter polysaccharide chains are added to numerous downstream serines and threonines of the Ywp1 core polypeptide. A sequence at the hydrophobic C-terminus triggers replacement of the final 22 amino acids with a membrane-embedded glycosylphosphatidylinositol (GPI) moiety, anchoring the glycoprotein to the plasma membrane. The GPI moiety is subsequently cleaved, and a remnant is linked to glucan chains that make up the matrix of the cell wall, thus anchoring mature Ywp1 to the wall by its new C-terminus, a feature shared by many fungal cell wall glycoproteins [11, 12].

Just downstream from the sole N-glycan attachment site of Ywp1 is a tribasic motif (triple arginine: -RRR-) separated by 8 amino acids from a dibasic motif (lysine-arginine: -KR-) that is further downstream; both of these sites are cleaved in mature Ywp1, and although the short intervening segment is lost, the upstream 11 kilodalton (kDa) “propeptide” remains tightly associated with the downstream core of Ywp1 [6]. (A schematic of these features of Ywp1 can be viewed in the Results section.) Propeptides are characteristic of a variety of secreted proteins in *C*. *albicans*, as well as proteins of many other organisms, and are typically involved in the proper folding or provisional inactivation of the downstream sequence, proper intramolecular disulfide formation, or independent signaling functions. The function of the Ywp1 propeptide is not known, but its sequence conservation and tight association with the downstream core segment suggest an important role.

Dibasic (-KR-) sites are a canonical recognition sequence for a large, well-characterized family of endoproteinases [13] related to the prototypical Kex2 of *Saccharomyces cerevisiae* [14, 15]; members have been documented in a wide variety of eukaryotes, including humans, where they have important roles in peptide hormone processing and the activation of bacterial toxins and pathogenic viruses [13, 16]. Target polypeptide cleavage typically takes place intracellularly, prior to secretion, in the late Golgi [17]. Deletion of Kex2 from *C*. *albicans* results in a strain that grows nearly as rapidly as the wild type, but is less virulent, and exhibits aberrant hyphal growth and aberrant processing of KR-terminated propeptides [18, 19], of which more than one hundred are predicted to exist in *C*. *albicans* [19, 20]. Notably, Kex2 is essential for the creation of Candidalysin, a lytic peptide fragment of Ece1 that is important for hyphal penetration of host epithelial barriers, and the first cytotoxic peptide toxin identified in a human fungal pathogen [21–23].

Initial characterization of Ywp1 from a strain that lacked Kex2 revealed the presence of a Ywp1 propeptide that was 12 kDa rather than 11 kDa, paradoxically suggesting cleavage at the -KR- site but not at the upstream -RRR- site [7]. Subsequent studies of wild type cells indicated that unnatural cleavage of Ywp1 was likely dependent on the very low pH (~2) attained *in vitro* by batch cultures, and likely performed by secreted acid proteases that also liberated much of the Ywp1 from the cell wall in a high molecular mass form; buffering of such cultures to a pH of 5–6 largely eliminated this observed “shedding” of Ywp1, however [6, 7]. Here it is shown that neither the 12 kDa nor the 11 kDa propeptide of Ywp1 is normally created in the absence of Kex2, yet the resulting Ywp1 molecule shows little alteration to its currently known characteristics. New insight is gained into possible direct or indirect roles of Kex2 in normal propeptide cleavage, for which few documented examples exist in *C*. *albicans*, as well as a possible distinction between the complex requirements for maturation and the performance of subsequent functions.

## Materials and methods

### Strains

Strains of *Candida albicans* used in this study are listed in Table 1.

**Table 1:** *Candida albicans* strain abbreviations and descriptions.

Strain designation	Brief description	Origin strain	Laboratory name(s)
**Wild type (wt) strains with respect to Kex2 and Ywp1**			
SC5314	Clinical isolate [24] used for first genome sequencing [25]		SC5314
CAI-4+CIp10	CAI-4 (*ura3/ura3* derivative of SC5314 [26]) with *URA3* restored by integration of the CIp10 plasmid at the *RP10* locus [27]	SC5314 … CAI-4	CAI-4+CIp10
BWP17	*arg4/arg4 his1/his1 ura3/ura3* [28]	SC5314	BWP17c [8]
**Other Kex2-possessing (*KEX2/KEX2*) strains**			
*ywp1/ywp1* strains	*ywp1*::*ARG4 / ywp1*::*URA3 HIS1* restored with *HIS1* [6, 7] *ywp1*::*ARG4 / ywp1*::*URA3 HIS1* restored with *pho100*::*GFP-HIS1*	BWP17	3L1 #6a
HomozygoticYwp165-Gfp	Ywp165-Gfp / Ywp165-Gfp [6]	BWP17	BF2d6
HomozygoticYwp520-Gfp	Ywp520-Gfp / Ywp520-Gfp [6]	BWP17	BG2d1
Ywp165-Gfp	*YWP1 / YWP1-GFP-URA3-GFP-YWP1* Cells secrete anchor-free Ywp165-Gfp (first stage of bifunctional transformation) [8]	BWP17	βWT1a βWT1a1a
**Kex2-negative (*kex2/kex2*) strains**			
CNA3	CNA3-1 and CNA3-2 (independent transformants) [18, 19] *kex2 / kex2 URA3 / ura3*	SC5314 … CAI-4	CNA3 (15.1) CNA3 (15.2)
CNA4	*kex2 / kex2 ura3 / ura3* [18]	CNA3-1	CNA4
k1 k2 k3 k4 k4a k4b	*YWP1 / YWP1-GFP-URA3* Cells secrete anchor-free Ywp165-Gfp	CNA4	1F3 1F7 1F1 1F4 1F4d 1F4e
k5 k6	*YWP1 / YWP1-GFP-URA3-GFP-YWP1* Cells secrete anchor-free Ywp165-Gfp (first stage of bifunctional transformation)	CNA4	βKX1b βKX1a βKX1a1
Ywp520-Gfp	*YWP1 / YWP1-GFP-URA3* Cells secrete anchor-free Ywp520-Gfp	CNA4	1G8
Ywp1-Gfp-Ywp1	*YWP1 / YWP1-GFP-YWP1* Cells make wall-anchored Ywp1-Gfp-Ywp1: Ywp1(aa1-165)-Gfp(aa1-233)-Ywp1(aa166-533) (second stage of bifunctional transformation)	CNA4	βKX1bΔU3 βKX1aΔU1a

### Culture media

Cells were grown in Minimal Medium 13 (MM13: 100 mM glucose, 80 mM NH_4_Cl, 5 mM NaCl, 5 mM KH_2_PO_4_, 1 mM MgSO_4_, 10 μM biotin, 0.5 mM succinic acid, 0.2 mM CaCl_2_, 2 μM FeCl_3_, 1 μM ZnCl_2_, 0.2 μM MnCl_2_, 0.2 μM CuCl_2_ [7]) or MM13 buffered with 100 mM MES (morpholinoethanesulfonate) and 50–80 mM Bis-Tris (bis-hydroxyethylimino tris-hydroxymethyl methane), pH 5.9–6.3 (BMM13). Supplemental 1 mM arginine, 1 mM histidine and 0.5 mM uridine were included as necessary for auxotrophies. To stimulate *YWP1* expression, the initial phosphate concentration was reduced for most cultures to less than 2 mM, the amount that is normally assimilated by batch cultures of MM13 prior to growth cessation [7] (see S1 Note). Tween-80 (0.02%) was often added as a carrier to inhibit adsorptive loss of secreted Ywp1 [6]. Media were sterilized by 0.2 μm filtration. For surface growth, media were hardened in Petri plates with 2.0% agarose.

### Gene engineering

*Candida albicans* strain CNA4 (*kex2/kex2*, *ura3/ura3*) was engineered to secrete Gfp-tagged, anchor-free versions of Ywp1 (“Ywp165-Gfp” and “Ywp520-Gfp”) as well as wall-anchored “Ywp1-Gfp-Ywp1”, both as described previously [8]. Briefly, the *GFP* that was employed encoded “yeast enhanced Gfp” with codon optimization for *C*. *albicans* and the enhancing mutations S65G and S72A [29]. PCR-amplified *GFP-URA3* cassettes were targeted for insertion downstream from codon 165 or 520 of *YWP1*, to encode chimeric polypeptides with the N-terminal 165 or 520 amino acids of Ywp1 appended to the N-terminus of Gfp. Recombinants were selected for growth in the absence of exogenous uracil or uridine; of the 72 Ywp165-Gfp transformants, 11 were analyzed, and 10 were found to secrete fluorescent Gfp; all 10 of the Ywp520-Gfp transformants were analyzed, and 8 were found to secrete fluorescent Gfp. Strain CNA4 was also transfected with a bifunctional cassette designed to allow both C-terminal and internal insertion of Gfp into Ywp1 following a single transfection, in order to successively generate both secreted and wall-anchored versions of Gfp-tagged Ywp1. The plasmid template for this cassette is available from Addgene (www.addgene.org/72606/). A PCR-amplified *GFP-URA3-GFP* cassette, with a 199 bp segment of *GFP* (encoding amino acids 168–233) repeated downstream from *URA3*, was targeted for insertion downstream from codon 165 of *YWP1*, encoding secreted Ywp165-Gfp identical to the Ywp165-Gfp described above. Nineteen of the 24 transformants were found to secrete fluorescent Gfp constructs; 3 of those were plated on nutrient agar containing 5 mM 5-fluoroorotic acid (5-FOA) and 0.2 mM uridine to select for loss of the inserted *URA3*. For each selection, 4–6 survivors were analyzed by fluorescence microscopy, and all but one was found to have Gfp fluorescence in the cell wall. Fluorimetry of these showed no Gfp fluorescence in the culture medium. PCR analyses of genomic DNA confirmed that the *URA3* had been lost through recombination of the identical flanking *GFP* segments, which resulted in *GFP* bridging the upstream and downstream *YWP1* segments (codons 1–165 and 166–534) to form a single open reading frame encompassing all 534 codons of *YWP1* and codons 1–233 of *GFP* [8].

### Protein analysis

Production and analysis of Ywp1 and its derivatives were performed as described previously [6–8]. The single N-glycan of Ywp1 was enzymatically removed with peptide-N-glycanase F (PNGase F; New England Biolabs); similar results were found using N-Zyme Scientifics PNGase PRIME from Bulldog Bio. Sodium dodecyl sulfate polyacrylamide gel electrophoresis (SDS-PAGE) utilized the Tris/Tricine/chloride system of Schägger and von Jagow [30] (S1 Fig only) or the Tris/glycine/chloride system of Laemmli [31], as modified and described previously [6–8]. Visualization of in-gel Gfp fluorescence by 488 nm laser scanning utilized a GE Typhoon Trio flatbed scanner with its PMT set at 350 and emission detection at 510 nm. Protein was visualized after gel fixation with 40% ethanol / 10% acetic acid, staining in the same mixture containing 0.1% Coomassie Blue R-250, and destaining with 15% ethanol / 5% acetic acid; alternatively (for S1 Fig only), protein was stained with silver [32–34].

### Microscopy and flow cytometry

Differential interference contrast (DIC) and epifluorescence microscopy were performed with a Nikon Eclipse 80i microscope equipped with an oil immersion apochromatic 60× objective lens (numerical aperture 1.4), and two filter cubes (FITC Ex465-495 DM505 BA515-555 for Gfp, and TRITC Ex540/25 DM565 BA605/55 for Alexa Fluor 568). Immunolabeling utilized anti-β-1,2-mannotriose (mouse monoclonal G11.1 IgG_1_ [35]) and goat anti-mouse IgG (H+L) conjugated to Alexa Fluor 568 (Molecular Probes) applied to heat-fixed (60°C, pH 8, 10 min) cells in 50 mM Tris (tris-hydroxymethyl aminomethane), 10 mM EDTA, 10 mM NaCl, 10 mM KCl, 0.1% Nonidet P-40 (pH 8.0). Micrographs were captured with a DS-Ri1 camera and NIS-Elements BR Imaging Software (3.10 SP3 Hotfix2). Images were cropped, resized and in one instance digitally overlayed in Adobe Photoshop, but were not otherwise modified.

Flow cytometry was performed with a Becton-Dickinson BD AccuriC6 flow cytometer with BD AccuriC6 software (version 1.0.264.21) using slow sample uptake (14μl/min; 10μm core), a FSC-H threshold of 300,000 (S3A and S3B Fig) or 200,000 (S3C Fig) a 488 nm excitation laser, and a 533/30 FL1 emission filter. Cells were applied to the instrument in pH 8 buffer, and for each sample, data on 100,000 events were collected.

### Gfp fluorescence quantitation

Using a Photon Technology International (PTI) spectrofluorometer, measurement of Gfp fluorescence in the cell culture medium was easily performed: Culture supernatants were adjusted to pH 8 with 0.087 V of 2.5 M Tris / 25 mM EDTA, placed in a 1 cm fluorescence cuvette, excited with 468 nm light, and the orthogonal 510 or 511 nm emissions quantified with a PTI Model 814 Photomultiplier Detection System. (Gfp produced an emission peak at 510–511 nm, whereas other compounds in the cells and medium did not.) Most of the Gfp remaining in the cells was then extracted with buffered (pH 8) 1% SDS + 25 mM DTT at 50°C for 15 min, and similarly quantified by using the same settings of the fluorometer. Additional experiments allowed minor corrections to be made to the observed values for each culture: The SDS + DTT treatment was found by fluorimetry to diminish the Gfp fluorescence by about 18%, and flow cytometric analysis of the cells before and after the SDS + DTT treatment indicated that this treatment extracted only about 90% of the fluorescence from the cells, so the measured values were adjusted accordingly. In addition, Gfp-free parental strain BWP17 was subjected in parallel to each analysis, and the observed “background” values (due to 510–511 nm autofluorescence and fluorescent components such as flavins that were observed by microscopy to be present in the cytoplasm rather than the periplasm or wall) were subtracted from the values for each Gfp-producing strain. Separate calculations were made for the mean and median flow cytometric values for all events (100,000 per sample) as well as the mean and median for just singlet cells (gated on the diagonal population of FSC-A vs FSC-H plots, representing 36–83% of the total events), and the final percentages were found to be within 1% of the values reported in the Results section. Variations due to slight differences in pH, photobleaching, and photodetector drift were relatively small and therefore ignored.

## Results

### Ywp1 propeptide genesis and detection

Batch cultures of *Candida albicans* grown in unbuffered Minimal Medium 13 (MM13, with glucose as the carbon source and ammonium as the nitrogen source) attain a pH of around 2 at stationary phase. Supernatants of such cultures contain Ywp1, which is easily detectable by Western blotting or by protein staining of the 11 kDa propeptide of Ywp1 that is prominent following deglycosylation and denaturation prior to SDS-PAGE [7]. For a strain devoid of Kex2 (strain CNA3; *kex2/kex2*), the Ywp1 Western blot profile was found to be slightly altered, and the 11 kDa propeptide appeared to be replaced by a 12 kDa propeptide [7]. The 12 kDa propeptide presumably resulted from endoproteolytic cleavage at the unique dibasic (-KR-) site that is about 1 kDa downstream from the tribasic (-RRR-) site, the site of the cleavage that produces the 11 kDa propeptide [7]. Paradoxically, -KR- is the canonical recognition and cleavage site for Kex2, which is missing from the CNA3 strain, but other proteases of *C*. *albicans* can also have this activity even in the absence of Kex2 [18]. Like the 11 kDa propeptide, the 12 kDa propeptide could be visualized as an SDS-PAGE band only after digestion with PNGase F to remove its N-glycan; its SDS-PAGE mobility was strikingly diminished by reduction of its disulfide bond; and its quantity increased upon phosphate limitation during growth of the culture ([7] and S1 Fig), all consistent with its identity as a slightly extended version of the 11 kDa propeptide. Furthermore, addition of Pepstatin A (but not 3 other protease inhibitors) to the culture diminished the quantity of detectable 12 kDa propeptide ([7] and S1 Fig); Pepstatin A inhibits secreted acid proteases [36, 37], which conceivably act on Ywp1 to create the 12 kDa propeptide but not the 11 kDa propeptide in these cultures. Co-culture of the Kex2- Ywp1+ strain with Kex2+ Ywp1- strains had no effect on the 12 kDa propeptide, indicating that active secreted proteases that might have been inactive when produced by Kex2- cells were not affecting propeptide genesis when supplied exogenously from a Kex2+ strain (S1 Fig). Initial studies of Kex2+ and Kex2- cells were thus enigmatic and inconclusive about the details of Ywp1 polypeptide scission and propeptide creation.

**Fig 1:**
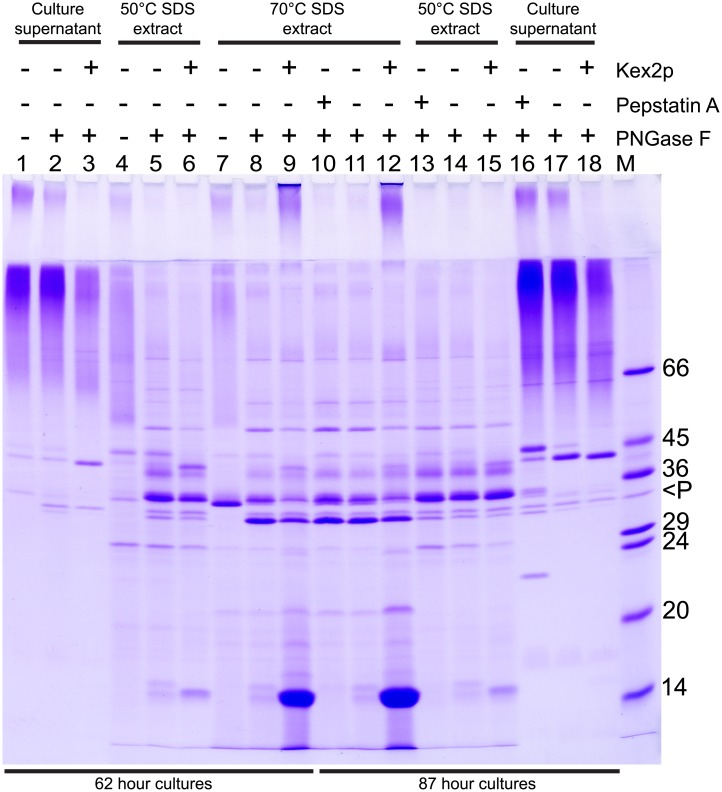
Analysis of Ywp1 cleavage in strains possessing or lacking Kex2. Cultures of wild type cells (+Kex2p: strain SC5314 in lanes 12, 15 and 18, and strain CAI-4+CIp10 in lanes 3, 6 and 9) were compared with *kex2/kex2* strain CNA3 15.1 (-Kex2p in all remaining lanes). Cells were grown for 62 or 87 hr (as indicated) in BMM13 that was buffered with 100 mM MES + 50 mM Bis-Tris (which allowed the pH to drop from 5.8 to only 5.4, rather than the usual 2.0); the initial phosphate concentration in all cultures was 0.3 mM (ultimately limiting), and the 87 hr culture also contained 0.02% Tween-80. One culture was given 20 μm Pepstatin A at 0 and 40 hr, as indicated. Culture supernatants, 50°C SDS extracts, and subsequent 70°C SDS extracts were concentrated by precipitation with ethanol (2 V). PNGase F digestions were performed in the presence of 10 mM DTT, and all samples were heated to 95°C in electrophoresis buffer containing SDS + DTT prior to SDS-PAGE and staining of the protein with Coomassie Blue. The masses (in kDa) of the marker proteins in lane M are shown on the right, with “<P” designating added PNGase F. The prominent reduced, deglycosylated, 11 kDa Ywp1 propeptide in lanes 9 and 12 migrated just behind the 14 kDa marker in this gel system; little or no propeptide was present in the samples from the *kex2/kex2* strain.

Subsequent studies of wild type cells revealed that buffering of the cultures to pH 5–6 greatly diminished the quantity of detectable 11 kDa propeptide in the surrounding medium ([6] and Fig 1, Lanes 3 and 18). (This mildly acidic pH range avoided the confounding downregulation of *YWP1* expression that would have occurred at neutral to alkaline pH.) Furthermore, treatment of washed cells with buffer at pH 1–2 did not then solubilize or extract Ywp1, suggesting that secreted acid proteases were cleaving wall-anchored Ywp1 in the more acidic cultures and causing fragments of Ywp1 to accumulate in the surrounding medium; the Ywp1 propeptide was never found free in the culture medium, however, without the core segment (or likely a fragment of it) also being present [6, 7]. Similar results have now been observed for the strain devoid of Kex2: Little or no propeptide (12 kDa or 11 kDa) was detected in supernatants from buffered cultures (Fig 1, Lanes 1, 2, 16 and 17). Cleavage of Ywp1 in unbuffered acidic cultures thus likely occurred at the dibasic -KR- site as well as other susceptible sites (but not the tribasic -RRR- site) well after secretion of Ywp1 from the cell and anchorage to the cell wall (in contrast to the expected intracellular location of Kex2-mediated propeptide cleavage).

An alternative method for extracting and visualizing the Ywp1 propeptide takes advantage of its strong association with the wall-anchored core of Ywp1. Treating intact yeast cells with SDS at 50°C extracts little of the Ywp1 propeptide, but a subsequent 70°C SDS treatment extracts nearly all of it ([6] and Fig 1, Lanes 6, 9, 12 and 15). Identical treatment of parallel buffered cultures of the strain devoid of Kex2 revealed that little propeptide was extracted at either 50°C or 70°C, and virtually none was extracted if Pepstatin had been added to the growing culture (Fig 1, Lanes 4, 5, 7, 8, 10, 11, 13 and 14). These cultures were grown for 62 or 87 hr; similar results were obtained for 24 and 39 hr cultures, suggesting no time-dependent creation or loss of this evidence. Thus, in a strain without Kex2, grown as yeast at pH 5–6, there appeared to be little or no cleavage of the Ywp1 polypeptide to generate a propeptide. This was not due to an absence of Ywp1 from the *kex2/kex2* cell wall, as indicated previously [7] and as demonstrated by the results below. Traces of Ywp1 propeptide presumably arose from Pepstatin-inhibitable secreted acid protease activity during culturing.

### Analysis of Gfp-tagged, secreted Ywp1

**Fig 2:**
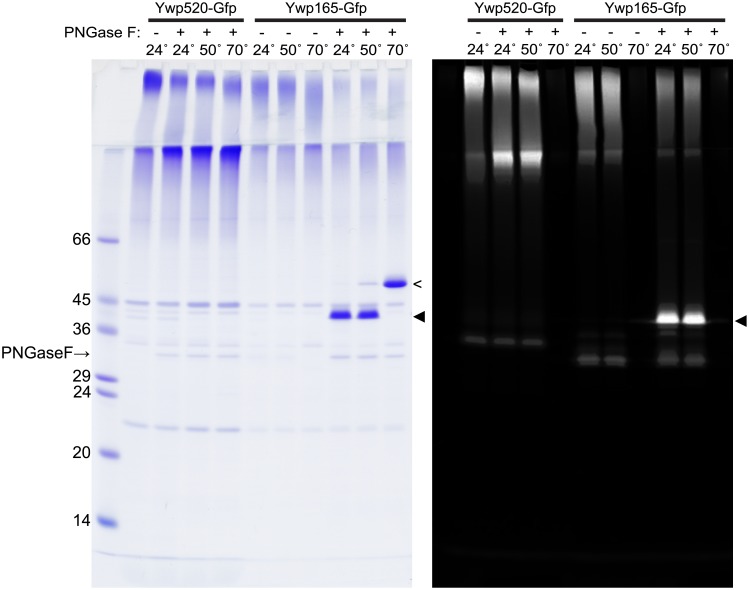
Analysis of anchor-free Ywp1-Gfp chimeras secreted from *kex2/kex2* cells. After 45–47 hr of growth in 30°C BMM13 with 0.3 mM phosphate, 0.02% Tween-80, 100 mM MES and 80 mM Bis-Tris (which allowed the pH to drop from 6.3 to 5.9), mannoproteins from alkalinized (to pH 8) culture supernatants were concentrated by ultrafiltration (10K MWCO), and digested (or mock digested) with PNGase F. Samples were then mixed with electrophoresis buffer containing SDS and DTT and either kept at room temperature (24°C) or heated for 5 min to 50°C or 70°C (as indicated) prior to SDS-PAGE. The gel was scanned for Gfp fluorescence (right panel) and subsequently stained for protein with Coomassie Blue (left panel). Markers are as in Fig 1. Deglycosylated Ywp165-Gfp migrates as a single band with native (fluorescent) Gfp (solid arrowhead) or denatured Gfp (open arrowhead); no propeptide is evident.

To investigate this propeptide deficiency further, anchor-free Ywp1-Gfp chimeras were engineered as described previously [6], except this time in a *kex2/kex2* strain (CNA4) to produce strain “k4”. Insertion of *GFP* into two positions of *YWP1* resulted in the synthesis and secretion of “Ywp165-Gfp” and “Ywp520-Gfp”, with 165 or 520 N-terminal amino acids of Ywp1 appended to the N-teminus of Gfp. As from wild type cells [6], these anchor-free chimeras were secreted from the *kex2/kex2* cells into the culture medium, where they were easily harvested by precipitation or ultrafiltration; as from wild type cells, they comprised the predominant protein species in the culture medium. Analysis of the chimeras by SDS-PAGE (visualizing the Gfp by fluorescence and, subsequently, the total protein by dye binding) revealed the following (Fig 2): Like these proteins from wild type cells, the Gfp remained fluorescent in SDS up to 50°C, but denatured and lost its fluorescence at 70°C; enzymatic removal of the N-glycan with PNGase F increased the mobility of Ywp520-Gfp, but it remained a large, poorly-resolved species, presumably because of the extensive and nonuniform O-glycan appendages on its 77.4 kDa polypeptide core; PNGase F digestion greatly increased the mobility of Ywp165-Gfp, creating two distinct bands commensurate with polypeptide length and whether the Gfp moiety was native (fluorescent) at ≤50°C or denatured (and nonfluorescent) at 70°C; unlike Ywp165-Gfp and Ywp520-Gfp from wild type cells, neither of these chimeras from *kex2/kex2* cells showed a propeptide band (11 kDa or 12 kDa) after deglycosylation and heating to ≥50°C to dissociate the propeptide from the core of Ywp1, nor did heating to ≥50°C without deglycosylation generate any well-defined Ywp-Gfp bands. Thus, both chimeras from the *kex2/kex2* strain were N-glycosylated, but no propeptide (or other fragment) was generated by cleavage of the Ywp1 polypeptide. The fully denatured Ywp165-Gfp polypeptide from the Kex2- strain had a mobility that suggested it was 10–15 kDa larger than the largest Ywp165-Gfp polypeptide from the Kex2+ strain (again indicating no cleavage at the dibasic or tribasic site).

Several independent transformants were similarly analyzed, and gave the same results as in Fig 2. (Analyses of three additional *kex2/kex2* Ywp165-Gfp secreters are shown as “k1”, “k2” and “k3” in S2 Fig) Additional experimentation with two subclones of strain “k4” showed denaturation of most but not all of the Gfp molecules by SDS at the intermediate temperature of 60°C, suggesting stochasticity rather than a firm threshold for thermal denaturation; heating to 95°C to ensure full denaturation of the polypeptide gave the same result as heating to 70°C (S2 Fig), revealing anomalously low mobility for this 42.7 kDa polypeptide relative to the 45 kDa and 66 kDa markers. Enzymatic removal of the N-glycan was found to be less efficient for native chimeras from the *kex2/kex2* strains than from wild type strains, remaining incomplete under non-denaturing conditions (Fig 2); prior denaturation of the chimeras (with SDS + DTT at 50°C) was found to allow complete removal of the N-glycan under the same digestion conditions (S2 Fig), suggesting a less accessible glycan-cleavage site if there is no propeptide cleavage.

### Insertion of Gfp into wall-anchored Ywp1

**Fig 3:**
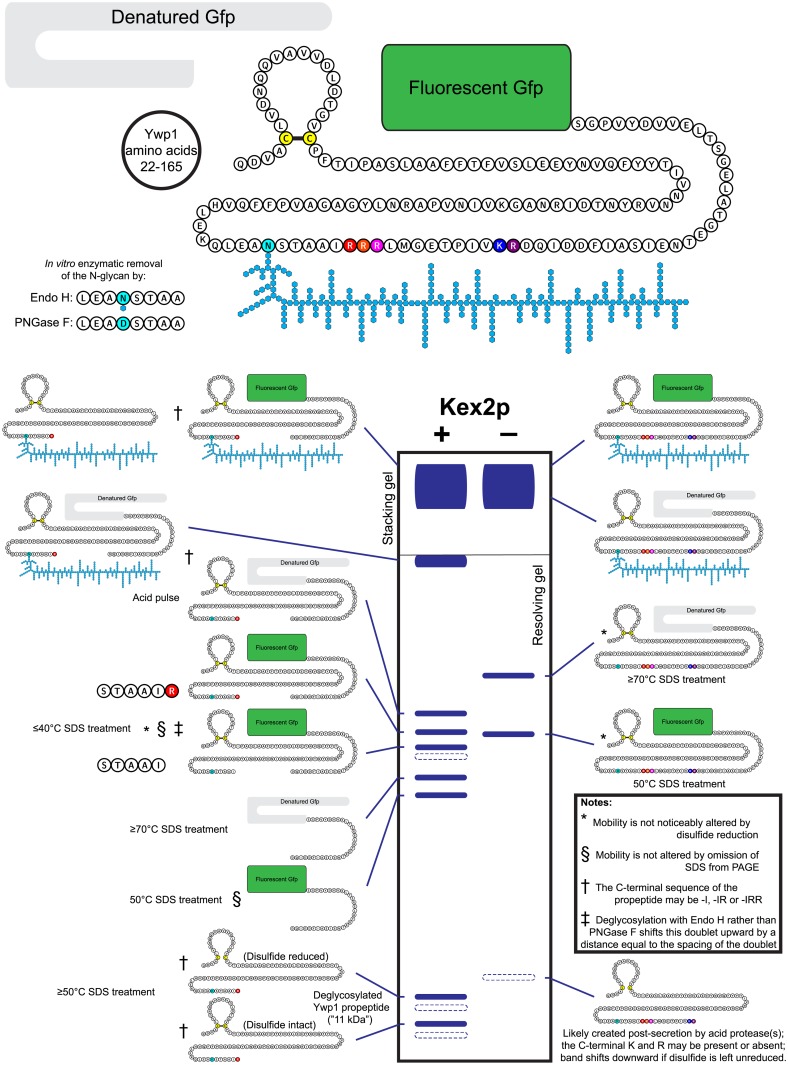
Schematic diagram of secreted Ywp165-Gfp and its various forms and derivatives resolved by SDS-PAGE. Components of the glycoprotein diagram are not drawn to scale; their conformations and spatial relationships were arbitrarily arranged here for compactness and clarity, and do not reflect any known or hypothesized arrangement. The Ywp1 amino acid chain was created using Protter, an online illustration tool [38]. The idealized schematic of the Coomassie Blue stained Tris/glycine/chloride gel shows the relative mobilities of the various proteins from strains possessing or lacking Kex2, as shown in previous publications [6–8] and previous figures in this paper. The dotted bands tend to appear and increase only upon prolonged storage, and are likely a result of low-level proteolytic degradative activity. The mobilities and relative positions of some bands may be different in alternative SDS-PAGE buffer systems [6].

The *kex2/kex2* strain (CNA4) was also transformed with a bifunctional cassette [8] that not only generated the secreted Ywp165-Gfp chimera, but also gave rise to strains with Gfp inserted into wall-anchored Ywp1 (after looping out and loss of the genomically integrated *URA3*), as described more fully below. Analyses of two independent transformants (“k5” and “k6”) are shown in S2 Fig, and demonstrate the same properties for their secreted Ywp165-Gfp as described above (and also further validate the first stage of the bifunctional cassette transformation). A concurrent analysis is shown in S2 Fig for Ywp165-Gfp from wild type cells that were transformed in parallel with the bifunctional cassette; for these Kex2+ and Kex2- strains (“wt” and “k6”), the figure directly compares the mobilities of the glycosylated and deglycosylated chimera polypeptides after the various experimental treatments to visualize the native and denatured Gfp and the associated and dissociated propeptide. It also shows the more efficient deglycosylation of the chimeric polypeptides from the wild type cells than from the *kex2/kex2* cells. A schematic diagram detailing Ywp165-Gfp and the various electrophoretically resolved bands is shown in Fig 3. The second stage of the transformation created tagged versions of Ywp1 that were analyzed microscopically (see below).

### Microscopic analysis of cells with Gfp-tagged Ywp1

**Fig 4:**
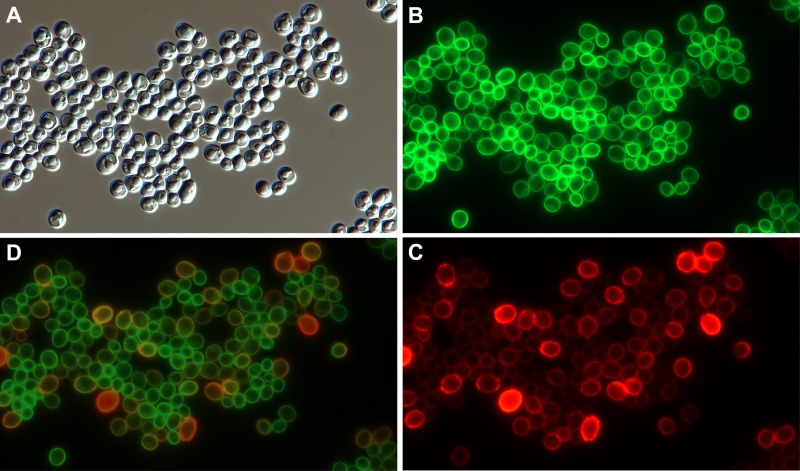
Localization of Gfp embedded in Ywp1 in *kex2/kex2* cells. Cells were grown on the surface of 2% agarose containing BMM13 with only 0.1 mM phosphate and buffered with 100 mM MES + 80 mM Bis-Tris (pH 6.3). After washing with pH 8 buffer, cells were fixed for 10 min at 60°C, then labeled with anti-β-1,2-mannotriose (MAb G11.1) and red Alexa Fluor 568-conjugated secondary antibody. (A) Differential interference contrast image; (B) Gfp fluorescence; (C) immunolocalization of β-1,2-mannotriose epitope; (D) digital overlay of panels B and C. Actual width of each image: 128 μm.

One of the derived Kex2- strains with Gfp inserted between amino acids 165 and 166 of wall-anchored Ywp1 is shown in Fig 4. As in Kex2+ cells [8], the Ywp1-Gfp-Ywp1 appears to accumulate in the cell wall. These cells were grown with limited phosphate, so expression of *YWP1* and production of Ywp1-Gfp-Ywp1 increased as the cells sensed this limitation, resulting in a range of Gfp fluorescence intensities. The absence of propeptide cleavage thus does not block transport of Ywp1-Gfp-Ywp1 to the cell wall. The cells were also labeled with a monoclonal antibody that binds to phosphodiester-linked β-1,2-mannotriose present on Ywp1 and other mannoproteins of the cell wall and visualized using a red fluorochrome-conjugated secondary antibody; the mannotriose epitope becomes scarcer as the cells are starved for phosphate, resulting in an inverse correlation with the quantity of Ywp1-Gfp-Ywp1 in the cell wall (Fig 4). The two signals thus provide complementary reflections of the history of phosphate limitation of each Kex2- cell, as was previously observed for Kex2+ cells [8]. The position of the Gfp within the thickness of cell wall is unknown, but based on previous studies [8], is unlikely to be on the outer surface or accessible to external antibodies; spatial separation between the red and green signals is visible in some cells in Fig 4, possibly accentuated by antibody bridging between the red fluorochrome and the mannotriose epitope, some of which must be exposed on the outer surface of the cell wall. Therefore, as assessed crudely by light microscopy, Kex2 status does not seem to markedly alter the position of Ywp1-Gfp-Ywp1 in the cell wall.

**Fig 5:**
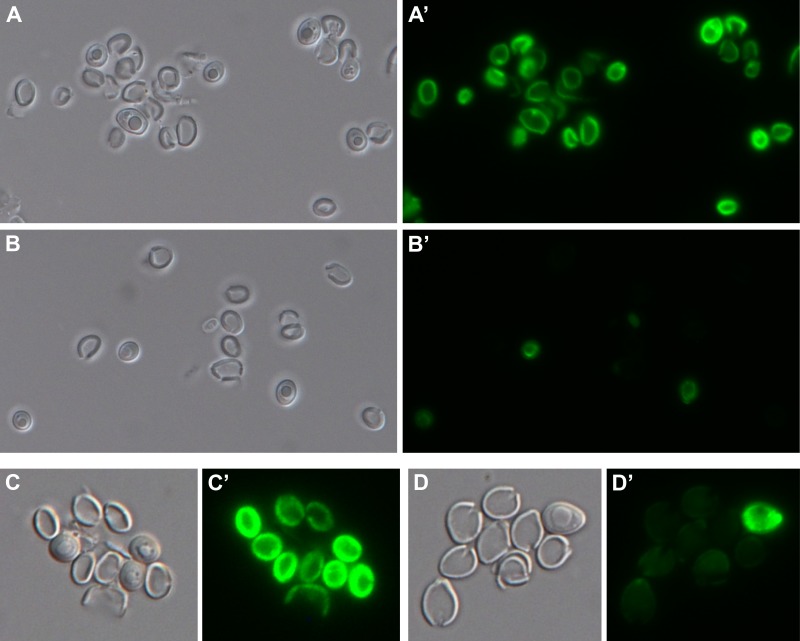
Wall anchorage of Gfp embedded in Ywp1 in *kex2/kex2* cells. Cells were grown as in Fig 2, washed with pH 8 buffer (100 mM Tris + 20 mM EDTA), and mechanically broken by vortex mixing with glass beads prior to extraction with 1% SDS + 20 mM DTT at 50°C (A, B) or 8 M urea + 0.5% CHAPS at room temperature (C, D). B, D: Strain that secretes Ywp165-Gfp. A, C: Offspring strain that synthesizes Ywp1-Gfp-Ywp1. A-D: Differential interference contrast images; A’-D’: Gfp fluorescence. Actual width of each image: A, B: 77 μm; C, D: 26 μm.

Microscopic analysis of some preparations of cells secreting Ywp165-Gfp revealed that not all of the Gfp fluorescence had left the cells, with some of it still visible in the cytoplasm (presumably in transit, or misfolded or aggregated, and thus possibly targeted for degradation) and much more of it present in the periplasm or wall matrix (two domains indistinguishable by conventional light microscopy). To determine if the Ywp1-Gfp-Ywp1 was indeed covalently anchored to the wall matrix of the *kex2/kex2* cells, the cell walls were mechanically ruptured, then extracted under conditions that should remove most noncovalently-bound proteins without diminishing the Gfp fluorescence (here, either 1% SDS + 20 mM DTT at 50°C, or 8 M urea + 0.5% CHAPS at room temperature). Fig 5 shows that fluorescent Ywp1-Gfp-Ywp1 indeed remained associated with the broken, extracted cell walls, whereas Ywp165-Gfp did not (and may therefore have been periplasmic or loosely associated with the wall). Cells in the preparation that were not broken retained more Gfp fluorescence, presumably because of inefficient prior passage through the intact cell wall. The reason for the lack of complete egress of Ywp165-Gfp from the living cells, however, is not known.

### Quantitative estimates of maturation and transport

To investigate whether Kex2-dependent Ywp1 cleavage might affect passage of Ywp165-Gfp through the cell wall into the surrounding medium, a full accounting of the detectable Gfp fluorescence was attempted for several cultures grown simultaneously in BMM13 with low phosphate and 0.02% Tween-80 to prevent adsorptive losses [6]. Cultures were assayed after shaking at 30°C for 50 hr and again after 78 hr, giving equivalent results. Gfp fluorescence in culture supernatants and cell extracts was quantified by fluorimetry, while that in the cells themselves was quantified by flow cytometry (S3 Fig). This accounting was subject to a multitude of minor uncertainties that were largely corrected for, as described in the Materials and Methods section, resulting in best estimates. Under this set of growth conditions, the proportion of synthesized Ywp165-Gfp that passed into the culture medium from a Kex2+ strain (“wt” in S2 Fig) was 85%, and from a Kex2- strain (“k4b” in S2 Fig) was 90%, with most of the remainder likely remaining, for unknown reasons, in the periplasm. The lack of Ywp1 cleavage (propeptide genesis) thus had a minor effect if any on this egress. Two homozygotic strains (with *GFP* inserted into both *YWP1* alleles rather than just one) that are Kex2+ were also assayed in parallel: Ywp165-Gfp (from strain BF2d6) showed 92% passage into the culture medium, while Ywp520-Gfp (from strain BG2d1) showed only 73% passage, presumably because folding, transport or egress of the long, O-glycosylated core was inhibited by its greater size, or because of additional interactions with components of the periplasm or wall.

As from Kex2+ cells [8], Ywp165-Gfp from Kex2- cells was much more abundant than the wall-anchored Ywp1-Gfp-Ywp1 in the directly derived (Δ*ura3* offspring) strains, possibly because the Gfp insertion had an inhibitory effect on downstream polypeptide folding, processing or transport. Flow cytometry revealed that after 90% of the Ywp165-Gfp was secreted into the medium, the cells still possessed more Gfp than did the Ywp1-Gfp-Ywp1 cells (see S3 Fig Panel C). Freezing and thawing of the cells liberated little of the remaining Ywp165-Gfp, but light digestion with β-1,3-glucanase + DTT followed by freezing and thawing allowed most of it to escape (S3 Fig), even though the cells appeared by light microscopy to still be intact. Little of the Ywp1-Gfp-Ywp1 was liberated under these conditions, however, again demonstrating its wall anchorage, despite the lack of propeptide cleavage (S3 Fig).

## Discussion

### Effects of propeptide cleavage

In the absence of Kex2, the intracellular polypeptide scission that normally creates the propeptide of Ywp1 does not occur. Nevertheless, Ywp1 appears to follow its normal biosynthetic and transport pathway, ultimately becoming a complex glycoprotein anchored within the cell wall of *C*. *albicans*. Note that proprotein cleavage does not affect the continued *presence* of the 11 kDa propeptide, which normally remains strongly associated with the Ywp1 core after cleavage [6]. Whether an absence of propeptide cleavage alters the amount of Ywp1 that normally accumulates in the wall is unknown, but quantities appear similar on the basis of immunodetection of the core epitopes by Western blotting and by visualization of a 12 kDa propeptide that is presumably generated artificially by extracellular acid proteases [7]. Positional alterations of Ywp1 in the cell wall have not been resolved by light microscopy, and propeptide cleavage has no obvious effect on the passage of anchor-free Ywp1 through the cell wall into the culture medium; the latter has been examined under a limited set of conditions *in vitro*, however, and little is known about how phosphate limitation and growth stages affect the glycan appendages of Ywp1 or the relevant structure or permeability of the cell wall. Insertion of a tandem hemagglutinin epitope into the Ywp1 core just 9 amino acids from the dibasic propeptide cleavage site was previously found to interfere with both propeptide cleavage and N-glycan addition [8]; wall anchorage was nevertheless still observed, indicating that these features are not essential for this component of the posttranslational journey.

Also unknown is whether a lack of propeptide cleavage affects the functions or activities of Ywp1. Wall-anchored Ywp1 normally has an anti-adhesive effect, and *kex2/kex2* strains (possessing Ywp1 with uncleaved propeptide) exhibit low yeast-form adhesivity (like their parent strain SC5314); a *kex2/kex2 ywp1/ywp1* strain, if found to be more adhesive, would suggest that propeptide cleavage is unnecessary for the anti-adhesive effect of Ywp1. Such a strain has not yet been created, however, and screening efforts have been unsuccessful in finding a variant of strain “k4” with spontaneous conversion of its single *YWP1* allele to *YWP1-GFP-URA3* (which would eliminate its anti-adhesive wall-anchored Ywp1, but double the amount of its ineffective, anchor-free version). Also remaining to be investigated is the possibility that Ywp1 without a cleaved propeptide still contributes to cell wall epitope masking, the only other known phenotype of Ywp1 [8]. Even though the cleaved propeptide remains associated with the wall-anchored core of Ywp1, the cleavage results in an observable change in the ease with which the nearby N-glycan can be enzymatically removed from the native protein *in vitro*, suggesting a conformational change conferred by cleavage.

### Role of Kex2 in propeptide genesis

The tribasic -RRR- site and nearby dibasic -KR- site of Ywp1 are both normally cleaved to generate the 11 kDa propeptide, but neither site is cleaved when Kex2 is absent from the cell. The fate of the short intervening segment (-LMGETPIVKR-) in Kex2+ cells is unknown, but its low sequence conservation [6] suggests it is more likely to be a spacer than a bioactive peptide. The dibasic site of Ywp1 presents a canonical cleavage sequence for fungal Kex2 homologs, suggesting that Kex2 itself normally performs this cleavage of Ywp1 in *C*. *albicans*; the possibility that this cleavage is performed by a different endopeptidase that is dependent on Kex2 for its own activation, however, has not been ruled out.

Kex2 may also be responsible for non-canonical cleavage of the upstream tribasic (-RRR-) site of Ywp1, based on the following considerations: When R is substituted for K in the -KR- site of *S*. *cerevisiae* α-factor, Kex2 from *S*. *cerevisiae* (ScKex2) cleaves this -RR- site *in vivo* about half as efficiently as the -KR- site [39]. Kex2 from *C*. *albicans* (CaKex2) is assumed to have similar recognition specificity, since it can properly cleave α-factor and killer toxin in a *S*. *cerevisiae* strain that lacks its own Kex2 [18]. CaKex2 also cleaves the secreted aspartyl protease Sap2 precursor at its downstream -KR- site [18]. More recently, a study concluded that Kex2 is the major protease in the secretory pathway of *S*. *cerevisiae*, and further defined its recognition and preferred cleavage sites [40]: As part of a recombinant reporter construct designed to traverse the secretory pathway of *S*. *cerevisiae*, a pentapeptide library representing all possible amino acid sequences was assessed for its susceptibility to cleavage by Kex2 in both a wild type and a Kex2Δ strain [40]; this analysis confirmed the -KR- site preference for Kex2 cleavage, but also revealed a strong preference for -LXXR-. Cleavage by ScKex2 thus occurs preferentially on the C-terminal side of P1 in the sequence P4-P3-P2-P1, where P1 is R, P2 is K or R, and P4 is L (or other aliphatic amino acid). This is consistent with earlier observations of substantial specificity and cleavage beyond the canonical -KR- site [13, 41–43]. As an additional example, when the precursor of human parathyroid-hormone-related hormone is treated with a secreted, soluble version of ScKex2, preferential cleavage occurs at an -LRRR- site, with -KR- sites also exhibiting cleavage [44], further supporting this expanded specificity. The tribasic sequence of Ywp1 is preceded by isoleucine, suggesting that this -IRRR- site may indeed be cleaved by CaKex2 as efficiently as the nearby canonical -IVKR- dibasic site. Whether these cleavages are random or sequential is not known.

Of the many potential polypeptide substrates for Kex2 in *C*. *albicans*, only a few have been experimentally investigated or verified. The N-terminal sequences of purified exo-β-1,3-glucanase (Xog1) and 65 kDa mannoprotein (Mp65) from *C*. *albicans* were found to lie immediately downstream from predicted -KR- sites, implicating Kex2 cleavage of the proproteins at those sites [45, 46]. *C*. *albicans* has ten secreted aspartyl proteases (Saps), all of which have a predicted propeptide terminating in -KR (or -KK for Sap7) with Kex2 sensitivity [47], as has been demonstrated by immunoblotting for Sap2 [18]. Consistent with its activity in *S*. *cerevisiae*, *CaKEX2* is required for mating and α-factor production by *C*. *albicans*, strongly implicating CaKex2 in α-factor precursor cleavage [48, 49]. Recombinant, affinity-tagged CaKex2 produced by *C*. *albicans* was found to cleave bacterially-derived recombinant versions of eight predicted substrate proteins from *C*. *albicans*, namely the α-factor precursor, Ece1, Sun41, Tos1, Pga17, CA0365, CA1873 and CA2974; for unknown reasons, five predicted substrate proteins were found to remain uncleaved under the same conditions [20]. Recombinant, affinity-tagged ScKex2 produced by *Pichia pastoris* [50] was found to cleave all but one of the eight substrates cleaved by CaKex2 [20], suggesting that the activities or specificities of ScKex2 and CaKex2 are similar but not identical. Cleavage of a chromogenic test substrate revealed enzyme characteristics (pH optimum, inhibitor sensitivities, *etc*.) that are similar for ScKex2 and CaKex2 [20]. Finally, *in vivo* studies as well as *in vitro* studies using the recombinant ScKex2 and CaKex2 described above indicated that the α-mannosidase Mns1 of *C*. *albicans* undergoes Kex2-dependent cleavage at a non-canonical site, apparently at the C-terminal side of one of the two arginines in the sequence -EEVSRARDWIK- [51], reinforcing the notion that context as well as sequence are important for Kex2 cleavage efficiency.

The best characterized Kex2 substrate in *C*. *albicans* is Ece1, a 271 amino acid preproprotein with 7 dibasic -KR- sites (but no -KK-, -RK- or -RR- sites), all of which are cleaved by Kex2 *in vitro* and *in vivo* [20, 22, 23]. Interestingly, mass spectroscopic analysis of secreted Ece1 peptides from *C*. *albicans* also indicated significant amounts of Kex2-dependent cleavage on the C-terminal side of -IVAR, -LITR, -VSER and -LIKK sequences [23], consistent with the expanded -L/I/V-X-X-R- specificity noted above for ScKex2, and supporting the possibility that CaKex2 also cleaves the -IRRR- tribasic sequence of Ywp1 to generate the 11 kDa propeptide. Notably, although *C*. *albicans* and the closely related *C*. *dubliniensis* both have the -IRRR- sequence, Ywp1 orthologs in other *Candida* species and closely related genera have -(I or L)-(RKR or KRR)- at that position [6], including within it the canonical -KR- sequence, and thus further support the possibility of Kex2-dependent cleavage at that site.

### Contribution of other proteases

An intriguing characteristic of the secreted Ywp165-Gfp version of Ywp1 is that the end of the 11 kDa propeptide does not exactly match the expected Kex2 cleavage site, which would result in a C-terminus of -IRRR, but instead consists of approximately equal amounts of -I and -IR ends [6], suggesting that 2 or 3 of the arginines remaining after the putative Kex2 cleavage event are subsequently trimmed off. Such trimming is likely to be performed by Kex1, a carboxypeptidase that has recently been characterized in *C*. *albicans* [22, 23]. Kex1 was originally characterized in *S*. *cerevisiae* as a carboxypeptidase B-like activity that removes basic amino acids from the C-termini of α-factor and killer toxin following Kex2 cleavage [52–54]; the enzyme resides in a Golgi compartment along with Kex2 [55, 56], indicating that substrate cleavage by both enzymes likely occurs prior to secretion. The ortholog in *C*. *albicans* was found to be critical for removal of the C-terminal arginine of Candidalysin, one of the peptides of Ece1 that is generated by Kex2 cleavage of the Ece1 precursor [22, 23], and important as a lytic peptide for hyphal invasion of epithelia [21–23]. Following the Kex2 cleavages that generate the 32 amino acid immature Candidalysin peptide that terminates in -KR, Kex1 removes the C-terminal R to give the K-terminated mature Candidalysin that predominates in hyphal secretions; the functional significance of this trimming is not yet clear, however [22, 23]. Functional significance of such trimming is nonetheless apparent for the α mating pheromone of *C*. *albicans*, which terminates with -PGKR following Kex2 cleavage; a version without R (with just K on the C-terminus) is much less active than a version with neither K nor R [49]. In *S*. *cerevisiae*, following Kex2 cleavage, removal of the C-terminal R by Kex1 is important for the activity of α-factor and killer toxin [14, 57]; intriguingly, ScKex2 itself has a cleaved propeptide that terminates in -LFKR, and the continued presence of its C-terminal R has been found to be necessary for the full activity of Kex2 [58]. If CaKex1 indeed trims either 2 or 3 of the C-terminal arginines from the Ywp1 propeptide, then there may be functional significance to this removal as well as to the observed equivalence in quantity of the resulting -I and -IR versions of the propeptide. Carboxypeptidase B from porcine pancreas readily removes the R from the -IR version *in vitro* [6], arguing against the existence of a propeptide subpopulation that is somehow resistant to this trimming. Confirmation of this difference as a pre-secretion feature of the Ywp1 propeptide awaits analysis in a *kex1/kex1* strain.

The polypeptide scissions that generate the Ywp1 propeptide presumably occur in a late Golgi compartment, but no experimental evidence addresses the timing or location of these cleavages; the possibility that they occur primarily extracellularly seems unlikely, but has not been ruled out, especially in light of the existence of extracellular proteases with overlapping specificities. Secreted aspartyl proteases are thought to be responsible for all of the extracellular proteolytic activity of *C*. *albicans* [47]; some of these Saps, particularly the wall-anchored Sap9 and Sap10, have substrate specificities that overlap Kex2, raising the possibility of their acting extracellularly on Ywp1 [36, 59]. At their optimal pH, native wall-anchored Sap9 and Sap10 do not seem to act on the dibasic, tribasic or other sites of wall-anchored Ywp1; at a sub-optimal pH, recombinant Sap9 (anchor-free and produced by *Pichia pastoris*) appears to cleave the wall-anchored Ywp1 core at a non-basic site, but was not seen to do so at its optimal pH; and HA-tagged Ywp1 that is naturally shed into the culture medium appears unchanged in strains lacking Sap9 and Sap10 [36], all arguing against a significant role for these Saps in Ywp1 maturation. Additional cleavages, such as at the sole arginine of the Ywp1 core that is 58 amino acids from its mature N-terminus, would be more difficult to identify because of interference by attached O-glycans. The shedding of Ywp1 into the culture medium, prominent at strongly acidic pH but minimal at a more neutral pH, may result from cleavage of the Ywp1 core at sites distal from the propeptide cleavages and more proximal to the C-terminal wall anchor, since the resulting products have low mobility during SDS-PAGE [6, 7]; Saps with acidic pH optima (or other uncharacterized acid proteases) may thus be capable of inefficient cleavage of Ywp1 at multiple sites under certain conditions, perhaps in a manner similar to the cleavage of Msb2, a conditionally shed mucin that has protective, sensing and signaling activities [60, 61]. In a strain without Kex2, such activity in acidic cultures may slowly target the dibasic site of Ywp1, but not the tribasic site, generating the 12 kDa propeptide but not the 11 kDa propeptide. The reliance of all Saps on Kex2 for their own propeptide cleavage and enzymatic activation further complicates these interpretations, however. Unidentified extracellular carboxypeptidases may further trim the 12 kDa propeptide as well as the 11 kDa propeptide upon prolonged storage [6, 7]. More detailed examination of the potential role of Saps in the proprotein processing of Ece1 revealed little or no contribution from these extracellular proteases [21, 23], further supporting the predominance of propeptide generation from these substrates by intracellular Kex2 and not extracellular Saps.

The remarkably acidic state attained by batch cultures of *C*. *albicans* in unbuffered Medium 13 is likely a result of the high concentration of the fermentable sugar glucose as the only source of carbon in this medium, an effect that has long been recognized in similar culture systems (for example, [62, 63]). For perspective, commensal and pathogenic *C*. *albicans* cells likely never experience such acidic environments in humans, except if passing through the stomach. Blood and most tissues are better buffered and have a pH value near neutrality, although the vaginal epithelium is moderately acidic, with a pH as low as 4. Vagina-simulative medium for *C*. *albicans* culture has a pH of 4.2 [64], and numerous studies examining the effects of culture pH on the *C*. *albicans* transcriptome and proteome compare media buffered at pH 4 *vs*. pH 7 or 8 (for example, [65, 66]). Cultures with intermediate pH values (pH 3–5) have not been quantitatively examined for their ability to result in the loss of Ywp1 from the cell wall, but likely would show a continuum of quantities of shed Ywp1 in the culture medium, from the observed high quantities at pH 2 to low quantities at pH 6. This would not only be a function of the ambient pH, but also of the production, activation and secretion of proteases during the respective growth conditions. As mentioned above, secreted acid proteases of *C*. *albicans* may be largely or exclusively represented by the Sap family. Sap1-3 are most active at a pH of 3–4, but also show activity at pH 2; Sap4-6 are most active at a pH of 5, while Sap9 and Sap10 are active at pH 5–8 [36, 47], suggesting that Sap1-3 might be the best candidates for causing the gradual shedding of Ywp1 from cells in cultures that attain a pH of 2, a possibility that could easily be tested with *sap* knockout strains. Strains lacking Kex2 could be utilized to determine whether the same activity results in cleavage of the dibasic site of Ywp1 to generate the 12 kDa propeptide, even though this would not be physiologically relevant, since Kex2 in wild type strains likely cleaves this site (and likely also the nearby tribasic site) quite efficiently, presumably before Ywp1 becomes extracellular.

### Function of the Ywp1 propeptide

The absence of Ywp1 propeptide cleavage in a Kex2-negative strain has not revealed the function of the Ywp1 propeptide, but has constrained the possibilities. Without this cleavage, Ywp1 is still synthesized, glycosylated, secreted and anchored to the cell wall, suggesting that if the propeptide performs a chaperoning or folding function, it must do so without a free C-terminus and while still a collinear extension of the core sequence. This seems plausible in light of the observation that the cleaved propeptide also remains tightly associated with the Ywp1 core, and non-denaturing conditions under which it can be separated from the core have not been discovered [6]. If the propeptide has a conditionally activating or inactivating function, then conditions that dissociate it from the core may exist; such conditions need not be physicochemically harsh, however, as additional undiscovered enzymatic modifications, for example, might result in conformational changes that affect Ywp1 function. Perhaps noteworthy is that the single N-glycan of Ywp1, presumably a large, complex mannan, is attached near the C-terminus of what becomes the cleaved propeptide; dissociation of the propeptide from the Ywp1 core would thus result in loss of this glycan from the cell, which might diminish the masking of other wall components [8]. The propeptide also possesses two cysteines that form a readily-reversible disulfide bond [7], and as such may have a role in proper disulfide formation within the Ywp1 core, or subsequent intermolecular disulfide formation in the cell wall, an important aspect of mature wall structure [67]. How these possibilities might be facilitated by severing the N-terminal 11 kDa of Ywp1 is currently unknown; more definitive indications will likely come from engineered Ywp1 mutants that specifically target these features.

## Supporting information

S1 Note Batch culture limitations. (PDF)

S1 Fig Accumulation of Ywp1 propeptide in the unbuffered culture medium of *Candida albicans* strains possessing or lacking Kex2. Cultures of wild type (SC5314) and *kex2/kex2* (CNA3) *Candida albicans* were grown in unbuffered MM13 with a phosphate concentration that was in surplus (initially 5 mM; the four cultures in Panel B marked with asterisks) or limiting (initially 0.2 mM) in shaking flasks at 30°C for 72–74 hr, giving a stationary phase OD_600_ of 6.0 ± 0.4. Culture supernatants were 0.2 μm-filtered and given Tris, EDTA and NaCl to 8 mM, 2 mM and 50 mM, respectively; mannoproteins were then precipitated with one volume of ethanol and dissolved in water to give 62.5x the original concentration. These samples were digested with PNGase F or left undigested, and heated to 95°C in SDS electrophoresis buffer prior to SDS-PAGE utilizing the Tris/Tricine/chloride system. Gels were silver stained, dried, and scanned. Two samples (in panel A) were given 20 mM DTT during PNGase F digestion and during heating with SDS; disulfides were otherwise left unreduced. Panel B: Three of the eight parallel cultures were supplied with periodic, fresh aliquots of protease inhibitor: 100 μM AEBSF, 10 μM Pepstatin A, or 10 μM Leupeptin + 25 μM TLCK were added at 0, 8, 24, 48 and 72 hr. Panel C: 200 μM Pepstatin A was added once, at 6 hr, and a control received the same amount of DMSO (0.17%), the vehicle for the Pepstatin A. Two cultures were co-cultures (in a 9:1 starting ratio) of *kex2/kex2* (CNA3) and a *ywp1/ywp1* strain (strain 3L1 or strain #6a). Yellow dots overlie unidentified bands that exhibit upward shifts upon disulfide reduction, but are unaffected by PNGase F digestion, do not bind anti-Ywp1 antibodies, and are present in strains devoid of Ywp1. The unidentified band at the level of the cyan bar was unaffected by disulfide reduction or PNGase F digestion. All CNA3 cultures were transformant 15.1, except for the penultimate lane in Panel B, which was independent transformant 15.2. Some of the results in this figure were previously mentioned but not shown [7], and are therefore presented here with controls to provide more thorough documentation of the observations and conclusions.
(PDF)

S2 Fig Additional analyses of secreted Ywp1-Gfp chimeras. Samples were prepared and analyzed as for Fig 2, with the following exception: For all of the samples but those in the lower right two panels, mannoproteins were concentrated by ethanol precipitation rather than ultrafiltration. In those experiments, the alkalinized culture supernatants were added to the ethanol rather than the usual procedure of adding ethanol to the supernatants, briefly exposing some of the Ywp165-Gfp to a concentration of ethanol that was high enough to denature the polypeptide and cause an upward shift in the band to match the 70°C-denatured band. Strains “k1-3” are independent transformants that give the same results as strain “k4” in Fig 2; strains “k5-6” and “wt” are the *kex2/kex2* strain (CNA4) and the wild type strain (BWP17) that were transformed with the bifunctional cassette to create Ywp165-Gfp secreters; the latter was included to show the relative mobilities of the bands for which propeptide cleavage did occur: The freed 11 kDa propeptide (dot), the fluorescent Ywp165-Gfp polypeptide (double arrowhead), and the denatured polypeptide (open circle). A subclone of strain “k4” (“k4b”) was found to secrete ~1.3× more fluorescent Gfp than its siblings (*e*.*g*., “k4a”), but otherwise appeared similar (lower left panel); additional unused portions of these samples were run again after subjecting two of them to higher temperatures (lower middle panels). The lower right panels show the same samples used for Fig 2, except these were partially denatured with SDS + DTT (at 50°C) *prior* to digestion with PNGase F, which facilitated compete removal of the N-glycan. The solid and open arrowheads are positioned as in Fig 2.
(PDF)

S3 Fig Flow cytometric quantitation of residual Ywp165-Gfp and wall-anchored Ywp1-Gfp-Ywp1 before and after extractions. Cells were grown as in Fig 2 for 50 hr (A), 78 hr (B) or 77 hr (C). (A, B) Strains secreting Ywp165-Gfp possessed (βWT1a1a, a subclone of “wt” in S2 Fig) or lacked (1F4e = “k4b” in S2 Fig) Kex2, as indicated, and are compared to control strain BWP17 (Kex2+, Gfp-); cells were analyzed live or after extracting for 15 min with 50°C 1% SDS + 25 mM DTT; all events are shown (ungated). (C) The Kex2- strains in this panel synthesize Ywp165-Gfp (“k6” in S2 Fig) or wall-anchored Ywp1-Gfp-Ywp1 (offspring strain of “k6”); cells were analyzed live or after partial digestion with glucanase + DTT (“G’ase”: cells were incubated at 23°C for 7 hr and 4°C for 5 d in pH 8.1 100 mM Tris / 20 mM EDTA / 0.02% Pluronic F-127 / 20 mM DTT containing glucanase [recombinant, protease-free β-1,3-glucanase (Quantazyme *ylg*) from Quantum Biotechnologies] at 40 U / ml, followed by a freeze/thaw cycle (“F/T”); singlet cell data are shown (corresponding to the diagonal on a FSC-A vs FSC-H plot, with FSC values between 0.5 and 1.5×10^6^).
(PDF)
