# Supplementary material for: Propeptide genesis by Kex2-dependent cleavage of yeast wall protein 1 (Ywp1) of Candida albicans
Source: PLoS One. 2018 Nov 26;13(11):e0207955. doi: 10.1371/journal.pone.0207955 (PMC6258133; doi:10.1371/journal.pone.0207955)
Supplement: S1 Note — (PDF) [file pone.0207955.s001.pdf]

S1 Note

Batch culture limitations

Microbe cultures are often routinely grown in batches, in aerated, orbitally-shaking flasks. Initially, oxygen may be the limiting nutrient, as better aeration often leads to faster growth. When allowed to proceed to stationary phase, growth conceivably ceases because of a buildup of toxic metabolites or because an essential nutrient is used up and no longer available for continued growth. In *Candida albicans*, *YWP1* expression in such batch cultures is induced when phosphate becomes the limiting nutrient; the starting concentration of phosphate can thus determine the final cell mass and the time at which phosphate limitation is sensed and responded to. Yeast cultures are sometimes grown or maintained in a rich, partly-defined medium such as YPD (1% yeast extract, 2% peptone, 2% dextrose) or GYEP (0.3% yeast extract, 1% peptone, 2% dextrose). Early investigations [1] indicated that phosphate might become limiting during growth of *C. albicans* in GYEP batch cultures, but not YPD batch cultures, as *GFP* reporting the expression of *YWP1* or *PHO100* (an inducible acid phosphatase) was sometimes found to have been induced when stationary phase cells were examined.

Difco reports the phosphate composition of Bacto yeast extract and peptone as 3.270% and 0.445%, respectively, resulting in a final concentration of 1.50 mM in GYEP and 4.38 mM in YPD (3.44 mM from yeast extract and 0.94 mM from peptone). Since at least 2 mM phosphate is needed in Medium 13 to avoid phosphate limitation, GYEP may indeed be phosphate limited.

To determine what might be ultimately limiting the growth of *C. albicans* in <u>YPD</u> cultures, components of Medium 13 [1] were individually added. Only magnesium ion noticeably increased the final cell mass. Supplementing the starting culture with 1 mM magnesium ion was found to increase the final optical density by 20-40%. Adding magnesium to an unsupplemented YPD culture at stationary phase allowed additional growth to occur, suggesting that growth had not ceased because of a buildup of any growth inhibitor.

Similar results had previously been found for *Saccharomyces cerevisiae*: When grown in YEPD (0.5% yeast extract, 1% peptone, 20% dextrose) batch cultures, magnesium ion was found to be the limiting nutrient [2]. Difco reports the Mg composition of Bacto yeast extract and peptone as 0.075% and 0.007%, respectively, resulting in a final concentration of 0.18 mM in YEPD and 0.37 mM in YPD (0.31 mM from yeast extract and 0.06 mM from peptone). A direct analysis of YPD has indicated a magnesium concentration of 0.49 mM [3]. Thus, relative to the other components of the medium, this quantity of magnesium is evidently insufficient for maximal growth.

Upon supplementation of YPD with magnesium, a different essential nutrient may become limiting in batch cultures. In line with the GYEP observations mentioned above, this nutrient appears to be phosphate, as *YWP1* expression appears to be induced under these conditions. Thus, when studying cellular responses in batch culture, one should be aware of confounding starvation responses that may arise as a function of the initial composition of the medium, regardless of how rich it is.

Additional note of interest

Candida albicans can utilize ethanol as a carbon source, even as its sole carbon source. A preliminary experiment has indicated that ethanol can even be assimilated from the vapor phase by broth cultures. Specifically, cells were inoculated into a Petri plate containing carbon-free medium (Medium 13 without glucose) and placed in a chamber surrounded by a reservoir of water containing 1% ethanol (172 mM); the cells grew slowly, but ultimately attained an optical density comparable to that of complete Medium 13. The ethanol presumably steadily partitioned from the surrounding solution into the cell culture medium to support cell growth. There was little growth of control cultures surrounded by water without the ethanol. A separate set of experiments suggested significant mortality if the surrounding water contained 10% ethanol. Exemplifying what is already well known, volatile nutrients and catabolites can be difficult to account for, but can have significant effects on cells.

References

1. Granger BL, Flenniken ML, Davis DA, Mitchell AP, Cutler JE. Yeast wall protein 1 of Candida albicans. Microbiology (Reading, England). 2005;151(Pt 5):1631-44. Epub 2005/05/05. doi: 10.1099/mic.0.27663-0. PubMed PMID: 15870471.
2. Dombek KM, Ingram LO. Magnesium limitation and its role in apparent toxicity of ethanol during yeast fermentation. Applied and environmental microbiology. 1986;52(5):975-81. Epub 1986/11/01. PubMed PMID: 3539018; PubMed Central PMCID: PMCPMC239160.
3. Eide DJ, Clark S, Nair TM, Gehl M, Gribskov M, Guerinot ML, et al. Characterization of the yeast ionome: a genome-wide analysis of nutrient mineral and trace element homeostasis in Saccharomyces cerevisiae. Genome biology. 2005;6(9):R77. Epub 2005/09/20. doi: 10.1186/gb-2005-6-9-r77. PubMed PMID: 16168084; PubMed Central PMCID: PMCPMC1242212.
