# Supplementary figures and images for: Propeptide genesis by Kex2-dependent cleavage of yeast wall protein 1 (Ywp1) of Candida albicans
Source: PLoS One. 2018 Nov 26;13(11):e0207955. doi: 10.1371/journal.pone.0207955 (PMC6258133; doi:10.1371/journal.pone.0207955)

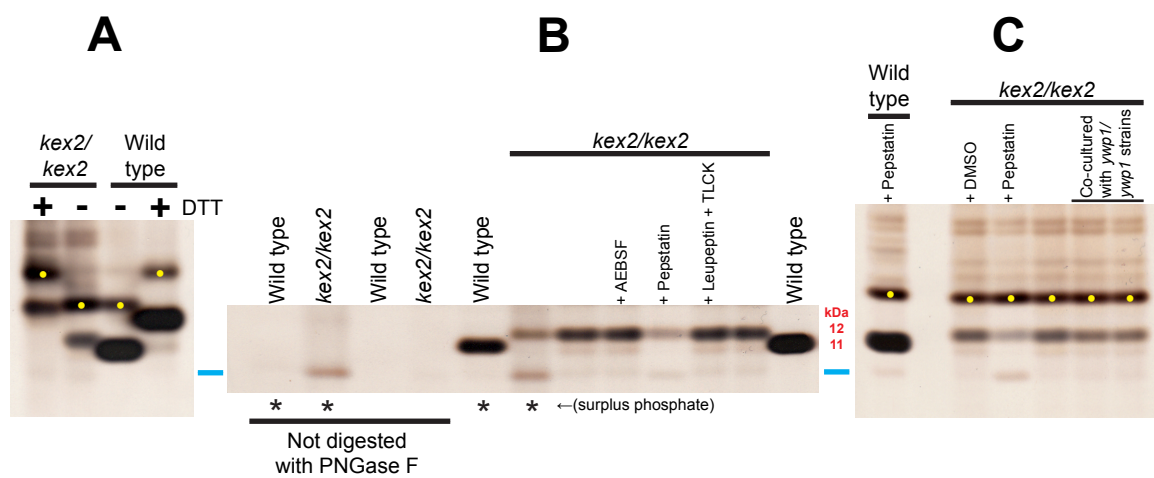

Supplement: S1 Fig — Cultures of wild type (SC5314) and kex2/kex2 (CNA3) Candida albicans were grown in unbuffered MM13 with a phosphate concentration that was in surplus (initially 5 mM; the four cultures in Panel B marked with asterisks) or limiting (initially 0.2 mM) in shaking flasks at 30°C for 72–74 hr, giving a stationary phase OD600 of 6.0 ± 0.4. Culture supernatants were 0.2 μm-filtered and given Tris, EDTA and NaCl to 8 mM, 2 mM and 50 mM, respectively; mannoproteins were then precipitated with one volume of ethanol and dissolved in water to give 62.5x the original concentration. These samples were digested with PNGase F or left undigested, and heated to 95°C in SDS electrophoresis buffer prior to SDS-PAGE utilizing the Tris/Tricine/chloride system. Gels were silver stained, dried, and scanned. Two samples (in panel A) were given 20 mM DTT during PNGase F digestion and during heating with SDS; disulfides were otherwise left unreduced. Panel B: Three of the eight parallel cultures were supplied with periodic, fresh aliquots of protease inhibitor: 100 μM AEBSF, 10 μM Pepstatin A, or 10 μM Leupeptin + 25 μM TLCK were added at 0, 8, 24, 48 and 72 hr. Panel C: 200 μM Pepstatin A was added once, at 6 hr, and a control received the same amount of DMSO (0.17%), the vehicle for the Pepstatin A. Two cultures were co-cultures (in a 9:1 starting ratio) of kex2/kex2 (CNA3) and a ywp1/ywp1 strain (strain 3L1 or strain #6a). Yellow dots overlie unidentified bands that exhibit upward shifts upon disulfide reduction, but are unaffected by PNGase F digestion, do not bind anti-Ywp1 antibodies, and are present in strains devoid of Ywp1. The unidentified band at the level of the cyan bar was unaffected by disulfide reduction or PNGase F digestion. All CNA3 cultures were transformant 15.1, except for the penultimate lane in Panel B, which was independent transformant 15.2. Some of the results in this figure were previously mentioned but not shown [7], and are therefore presented here with controls [file pone.0207955.s002.pdf]

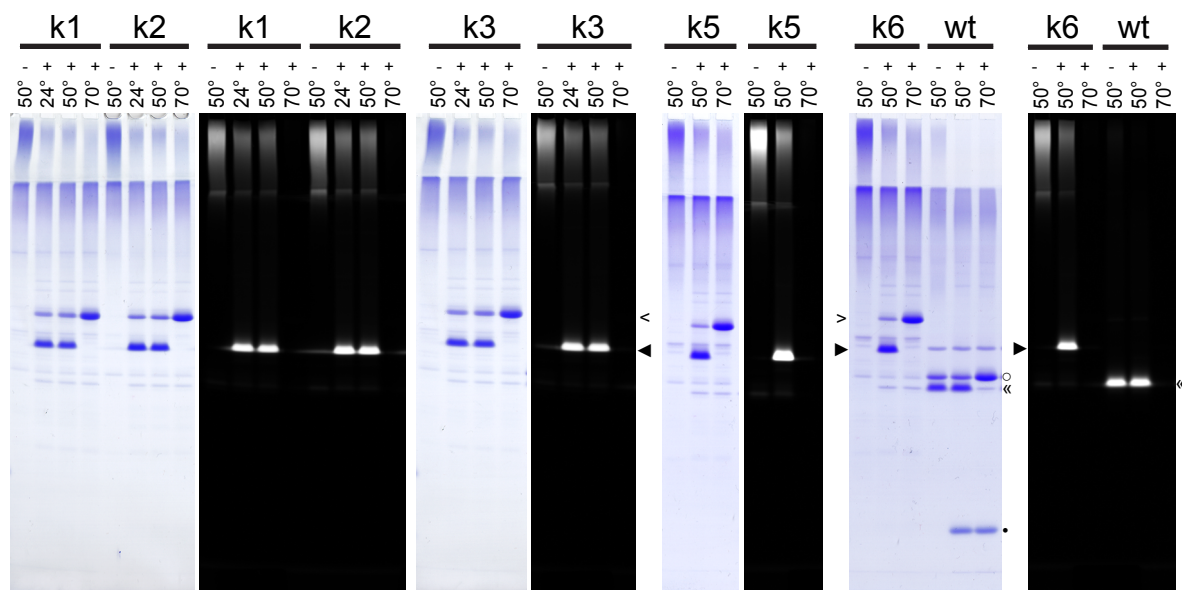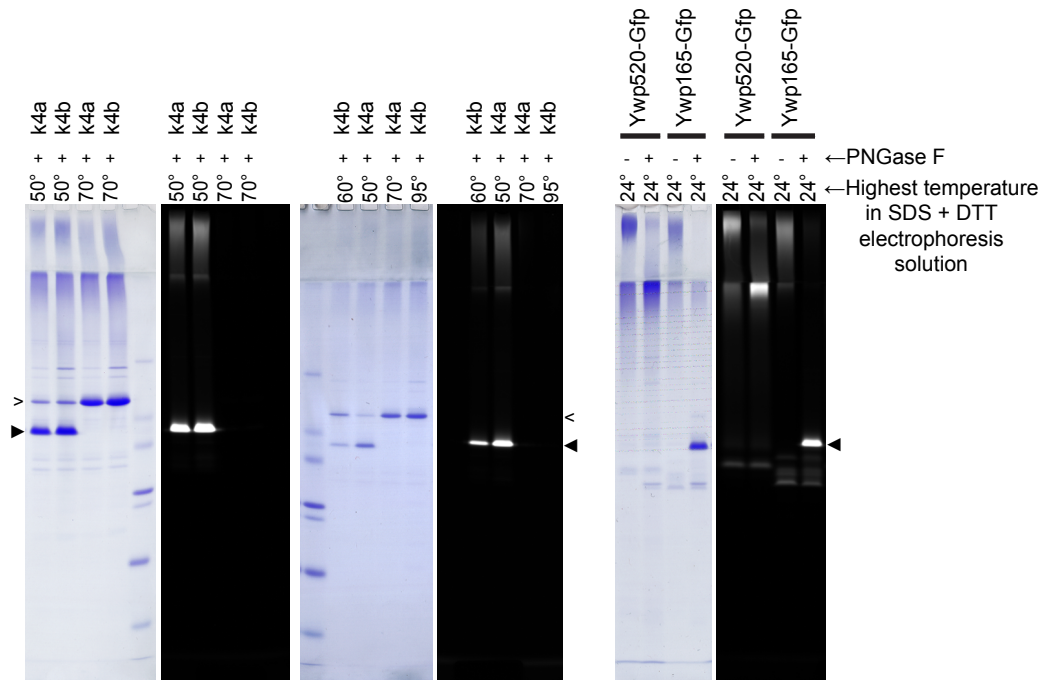

Supplement: S2 Fig — Samples were prepared and analyzed as for Fig 2, with the following exception: For all of the samples but those in the lower right two panels, mannoproteins were concentrated by ethanol precipitation rather than ultrafiltration. In those experiments, the alkalinized culture supernatants were added to the ethanol rather than the usual procedure of adding ethanol to the supernatants, briefly exposing some of the Ywp165-Gfp to a concentration of ethanol that was high enough to denature the polypeptide and cause an upward shift in the band to match the 70°C-denatured band. Strains “k1-3” are independent transformants that give the same results as strain “k4” in Fig 2; strains “k5-6” and “wt” are the kex2/kex2 strain (CNA4) and the wild type strain (BWP17) that were transformed with the bifunctional cassette to create Ywp165-Gfp secreters; the latter was included to show the relative mobilities of the bands for which propeptide cleavage did occur: The freed 11 kDa propeptide (dot), the fluorescent Ywp165-Gfp polypeptide (double arrowhead), and the denatured polypeptide (open circle). A subclone of strain “k4” (“k4b”) was found to secrete ~1.3× more fluorescent Gfp than its siblings (e.g., “k4a”), but otherwise appeared similar (lower left panel); additional unused portions of these samples were run again after subjecting two of them to higher temperatures (lower middle panels). The lower right panels show the same samples used for Fig 2, except these were partially denatured with SDS + DTT (at 50°C) prior to digestion with PNGase F, which facilitated compete removal of the N-glycan. The solid and open arrowheads are positioned as in Fig 2. (PDF) [file pone.0207955.s003.pdf]

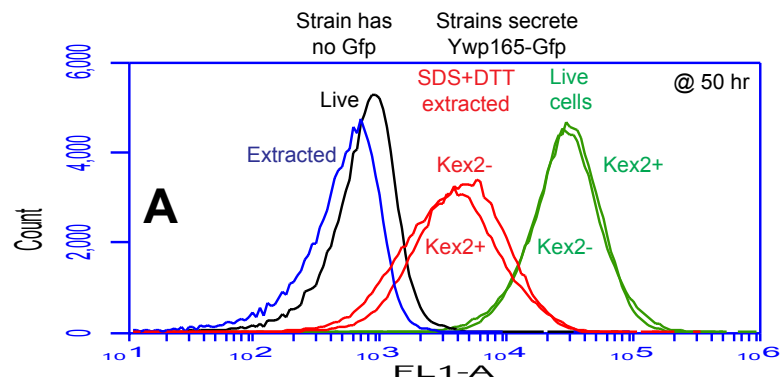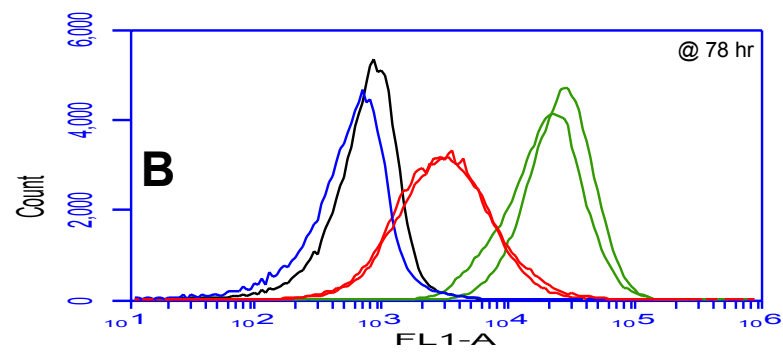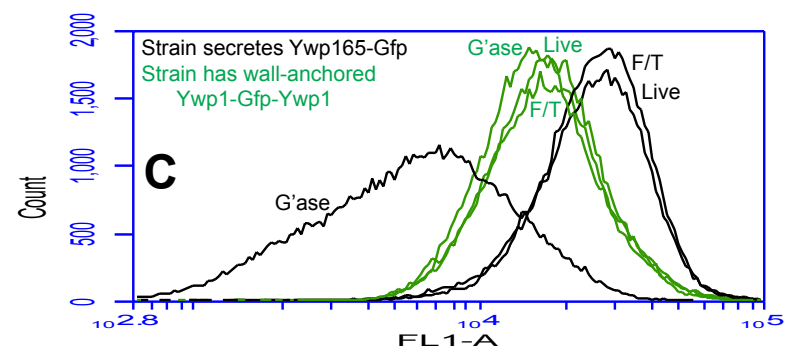

Supplement: S3 Fig — Cells were grown as in Fig 2 for 50 hr (A), 78 hr (B) or 77 hr (C). (A, B) Strains secreting Ywp165-Gfp possessed (βWT1a1a, a subclone of “wt” in S2 Fig) or lacked (1F4e = “k4b” in S2 Fig) Kex2, as indicated, and are compared to control strain BWP17 (Kex2+, Gfp-); cells were analyzed live or after extracting for 15 min with 50°C 1% SDS + 25 mM DTT; all events are shown (ungated). (C) The Kex2- strains in this panel synthesize Ywp165-Gfp (“k6” in S2 Fig) or wall-anchored Ywp1-Gfp-Ywp1 (offspring strain of “k6”); cells were analyzed live or after partial digestion with glucanase + DTT (“G’ase”: cells were incubated at 23°C for 7 hr and 4°C for 5 d in pH 8.1 100 mM Tris / 20 mM EDTA / 0.02% Pluronic F-127 / 20 mM DTT containing glucanase [recombinant, protease-free β-1,3-glucanase (Quantazyme ylg) from Quantum Biotechnologies] at 40 U / ml, followed by a freeze/thaw cycle (“F/T”); singlet cell data are shown (corresponding to the diagonal on a FSC-A vs FSC-H plot, with FSC values between 0.5 and 1.5×10^6^). (PDF) [file pone.0207955.s004.pdf]
